# Bio-Based Mulching Films and Soil Conditioners for Non-Irrigated Tomato Cultivation: Toward Plastic-Free and Water-Efficient Crop Production

**DOI:** 10.3390/ijms26209894

**Published:** 2025-10-11

**Authors:** Alessandro Sorze, Francesco Valentini, Tiziana Nardin, Roberto Larcher, Janine Bösing, Sebastian Hirschmüller, Andrea Dorigato, Alessandro Pegoretti

**Affiliations:** 1 Department of Industrial Engineering and INSTM Research Unit, University of Trento, via Sommarive 9, 38123 Trento, Italy; 2 Fondazione Edmund Mach—Technology Transfer Centre, via Edmund Mach 1, 38010 San Michele all’Adige, Italy; tiziana.nardin@fmach.it (T.N.);; 3 Department of Wood Technology and Construction, Technical University of Applied Sciences Rosenheim, Hochschulstraße 1, 83024 Rosenheim, Germany; janine.boesing@th-rosenheim.de; 4 Department of Research, Development and Transfer, Technical University of Applied Sciences Rosenheim, Hochschulstraße 1, 83024 Rosenheim, Germany

**Keywords:** xanthan, gelatine, degradable polymers, topsoil cover, soil conditioner, mulch, tomato, sustainability

## Abstract

This study examined the impact of different bio-based and biodegradable mulching films (TSCs) and soil conditioners (SCs) on plant productivity and fruit quality in a tomato cultivation trial under non-irrigated conditions. In particular, different TSCs were developed based on xanthan gum (XG) or gelatine (GEL) mixed with wood fibres (WFs), while SCs were produced using XG and cellulose fibres. A total of 72 plants of *Solanum lycopersicum var. cerasiforme* were planted. The yield and number of fruits were measured at harvest, followed by physico-chemical analyses, while plant root systems were examined at the end of the experimental period. The results highlighted that the GEL-based TSCs improved the total fruit yield compared to the control (+50% on average). Furthermore, improved fruit yield was also observed for the XG-based SCs when applied in the soil with a higher organic content. Overall, no significant differences in fruit quality (i.e., Brix degree, carotenoids, lutein and potassium content) and plant root system parameters were found for all the treatments applied. At the end of the test, it was noticed that GEL-based films substantially retained their consistency due to their greater density and thickness, while XG-based films were more disintegrated, indicating higher biodegradation.

## 1. Introduction

Inadequate soil conditions, such as insufficient water and nutrients, sub-optimal temperatures, and weed infestations, are an emerging problem in agriculture. These conditions limit crop growth and productivity, contributing to a global food challenge in the context of a rapidly expanding human population. [1,2,3]. This situation becomes even more critical during summer, when heat and drought stress are combined [4,5]. Specifically, tomato cultivation (*Solanum lycopersicum* L.), which is one of the most globally relevant crops due to its high nutritional value, is negatively affected by the combined effects of heat and water stress, leading to a potential yield reduction of up to 90% [6,7,8,9]. To address the rising food demand, different research studies have explored various strategies to alleviate these problems [10,11,12,13,14]. Mulching films are among the most promising approaches for improving soil properties and water use efficiency, ultimately enhancing crop growth [15,16,17,18]. These products reduce soil moisture evaporation, regulate soil temperature, inhibit weed growth, and may support agricultural practices such as drip irrigation and fertigation [19,20,21,22]. Currently, most mulching films are made of plastic (e.g., polyethylene and polypropylene) [16,23], covering over 128,000 km^2^ of agricultural land worldwide [24]. Despite the economic advantages associated with the use of plastic films, concerns about microplastic release from mechanical damages, UV degradation, and erosion by wind and rain are driving research towards more environmentally friendly alternatives [16,23,25,26]. To overcome these limitations, different research studies investigated biodegradable alternatives such as polyhydroxyalcanoates (PHAs), polybutylene adipate terephthalate (PBAT), poly(lactic acid) (PLA), starch, alginates, chitosan, proteins, or their combinations [27,28,29,30,31,32]. Moreover, the addition of organic fillers (e.g., wood fibres) has been reported to improve mechanical performance of bio-based mulching films [33,34]. Other studies have found that the water barrier properties of polysaccharide-based mulching films can be enhanced by adding cross-linking agents (e.g., citric acid) [35] or through chemical surface modification [36]. However, the combination of good mechanical strength, sufficient water barrier properties and low production costs remains challenging, compromising their commercial viability. Furthermore, some literary studies have found that the excessive use of mulch increases soil temperature, which decreases its ability to absorb nutrients, resulting in reduced plant growth [18,37].

Another promising approach to improve water use efficiency and enhance crop growth is the use of soil conditioners (SCs), i.e., products added to the planting soil to enhance its physical, chemical, mechanical, and water-regulating characteristics [38]. Several studies have shown that SCs based on superabsorbent hydrogel polymers (SAPs) can effectively reduce water consumption during irrigation, lower plant mortality rates and promote plant growth, helping to address the problems caused by drought and water shortage [39,40,41,42,43,44]. Specifically, these studies showed that adding less than 2% SAPs to the soil increased its water absorption capacity by up to 60%, and in some cases increased plant growth by up to 50%. However, traditional SCs are made of synthetic polymers like polyacrylamide, which have limited biodegradability and can release harmful by-products into the soil, posing risks to the environment and human health [45,46,47,48].

To address these challenges, our research groups have recently developed different bio-based and biodegradable composites for use as topsoil covers (TSCs, i.e., multifunctional mulching films aimed at providing a more favourable environment for plant growth) and SCs. In particular, we produced two types of TSCs: one based on xanthan gum (XG) cross-linked with citric acid (CA) or tannic acid (TA) and reinforced with wood fibres (WFs), and the other based on gelatine (GEL) cross-linked with TA and also reinforced with WFs. For SCs, mixtures of XG and cellulose fibres were produced. XG is a polysaccharide obtained from *Xanthomonas campestris* in aerobic conditions from sugar cane, maize or their derivatives [49,50,51], while GEL is a protein derived from collagen-containing animal scraps [52,53]. The specific formulations used in this work were derived from our previous works on the development and optimisation of these products [35,54,55,56,57,58,59]. For TSCs, cross-linking XG and GEL with CA or TA significantly improved their thermal, mechanical, and water-insoluble hydrogel properties. Furthermore, XG-based TSCs cross-linked with CA or TA had excellent water absorption (up to 1900%), strong vapour barrier and mechanical performance. The addition of WF provided good dimensional stability. Nursery trials confirmed their positive effects on plant growth and soil microbial diversity [57,58].

The aim of this work is to study these optimised TSC and SC formulations in tomato cultivation under non-irrigated conditions. Being very sensitive to heat and drought, tomato plants were particularly suitable for the purpose of this study, in which no irrigation was applied to the plants. In particular, the yield productivity of plants, the fruit quality and plants root system were studied and compared across the different treatments. To the best of our knowledge, this is the first study investigating the effect of XG- and GEL-based TSCs and SCs on tomato plant growth under non-irrigated conditions.

## 2. Results and Discussion

### 2.1. Evaluation of Yield Characteristic

Table 1 shows the total yield, the number of fruits grown per plant (NFT) and the relative yield values of the treated tomato plants compared with those of the untreated plants for each row.

Table 1 shows no statistically significant difference at the 0.05 level between the total yield and NFT values across the different treatments and the control. Moreover, the reported NFT values have a high standard error, due to large differences in fruit production between plants within the same sample group. For this reason, the relative yield values of the treated tomato plants, evaluated in comparison with the untreated plants, are also reported for each row. Indeed, an increase in yield compared to the control is evident for nearly all samples in row I. Specifically, tomato plants treated with TSC_GEL showed the highest yield, indicating that this TSC formulation was particularly effective in enhancing the production of the tomato plants. On the other hand, in rows II and III, the yield of most treated plants was lower than that of the control. This behaviour could be related to the high organic content of the soil in row I (as reported in Table 6), which, as extensively reported in the literature, strongly affects the soil water storage capability [60,61]. Therefore, it can be hypothesised that the addition of TSCs and SCs consisting of XG also benefits from the presence of a larger organic content in soil, allowing them to exploit their full potential.

**Figure 1:**
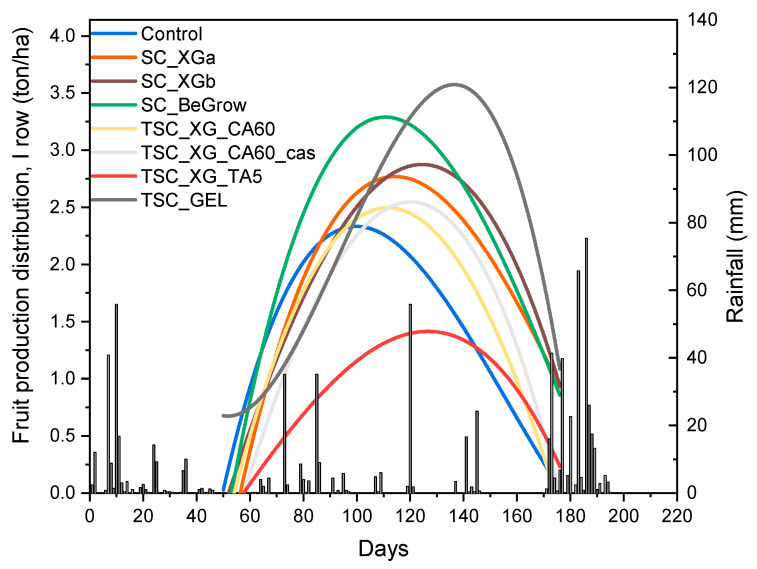
Fruit production distribution over time of the tomato plants in row I (full lines) correlated to the rainfall distribution (bar plot).

Figure 1 shows the distribution over time of the fruit productivity of plants in row I correlated with rainfall.

Figure 1 shows that, despite the similar curve profiles of all samples in the first months, the peak of the distribution curves of the treated plants is delayed in timing and increased in intensity, compared to that of the control. This appears to be related to rainfall distribution; specifically during the period of low rainfall, between days 100 and 160, the control plants showed a drop in productivity. On the other hand, the treated plants, which had higher water availability due to the presence of hydro-retentive TSCs or SCs, showed an extended production period, with an increased total fruit yield even under drought conditions.

### 2.2. Physico-Chemical Analysis of Tomato Fruits

Table 2 shows the physico-chemical characteristics of the fruits obtained from the different treated plants.

According to Table 2, the different treatments do not affect fruit quality at a statistical significance level of 0.05. In general, the dry matter content ranges from 7.7% to 8.3%, while the Brix degree varies from 6.53° to 7.32°. The lycopene and β-carotene contents vary from 143.0 mg/kg to 207.7 mg/kg and from 97.3 mg/kg to 138.7 mg/kg, respectively. The lutein amount ranges from 8.0 mg/kg to 13.4 mg/kg, while the potassium content varies from 2884.0 mg/kg to 3244.3 mg/kg. The results obtained are consistent with the physico-chemical characteristics of tomatoes reported in the literature [11,13,62].

### 2.3. Evaluation of Plant Root System

Table 3 shows the results of the investigation of the plant root system.

As reported in Table 3, there is no significant difference at the 0.05 level in the studied parameters of the root system among the treated tomato plants. In general, the root length and the stem diameter range from 32.6 cm to 34.8 cm and from 0.8 cm to 1.0 cm, respectively. The root:shoot ratio ranges from 0.27 to 0.41, while the root water content varies from 76.8% to 80.9%. These results are interesting because the literature reports that using mulch can sometimes reduce the root length, stem diameter, and root-to-shoot ratio due to excessive soil temperature increases and subsequent reductions in nutrient absorption [18,37].

**Figure 2:**
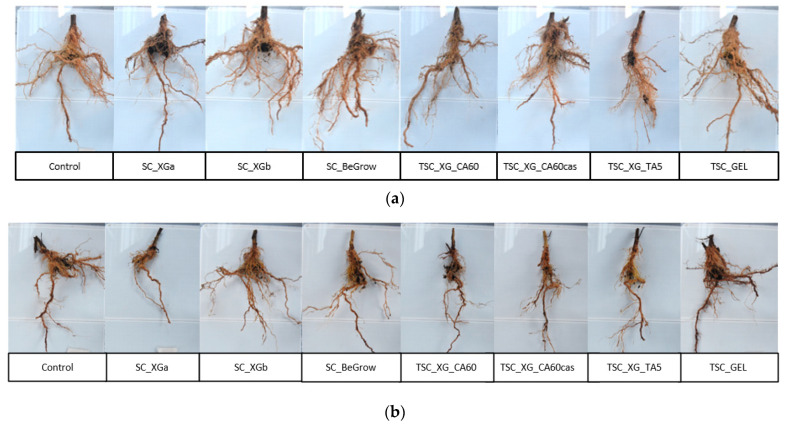

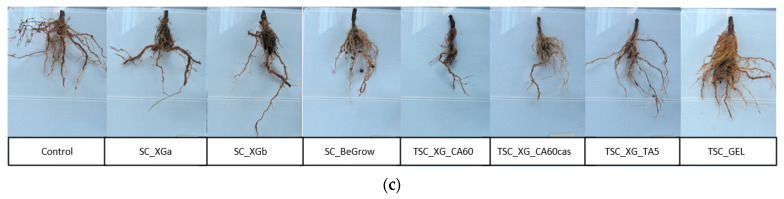
Representative images of the root system of the plants treated with different TSC and SCs samples: (**a**) row I, (**b**) row II and (**c**) row III.

For a better comparison of the results, in Figure 2a–c, representative images of plant root systems are reported.

Figure 2a–c show that root size and morphology are correlated with plant productivity (Table 1). Figure 2a shows that the roots in row I are larger in volume than those in the other rows. Moreover, by comparing the roots of the treated plants with those of the control sample, it is possible to notice that in row I, most of the treated plants are characterised by a greater volume of fine roots (which account for the absorption of water and nutrients of the plant [63,64]. Indeed, as reported in Table 1, in row I, most of the treatments have led to higher fruit productivity. On the other hand, in row II and row III, the roots of control are more developed than in most of the treated plants. These differences in root growth among the three rows may again be explained by the different organic contents in the soil, which may have influenced the performance of TSCs and SCs consisting of XG. The only exception is the TSC_GEL roots in row III, which are characterised by a greater volume of fine roots. This feature is reflected in the higher fruit productivity of this treatment, which showed good performance regardless of soil composition. Comparing the different treatments, it is interesting to notice that the SC_XGb sample (i.e., SC applied at the bottom of the pit) generally shows more developed roots (similar to the commercial product) with respect to SC_XGa (i.e., SC mixed with soil), likely because the SC_XGb treatment concentrates water locally rather than dispersing it throughout the soil. Regarding the three TSCs based on XG, it seems that the treatment with casein led to larger growth of fine roots in the first row (although this was not reflected in the yield results), while in the other rows, it is difficult to identify any difference between these three treatments.

### 2.4. Evaluation of Mulching Residues

In Table 4, the residual masses of the TSCs are reported.

**Figure 3:**
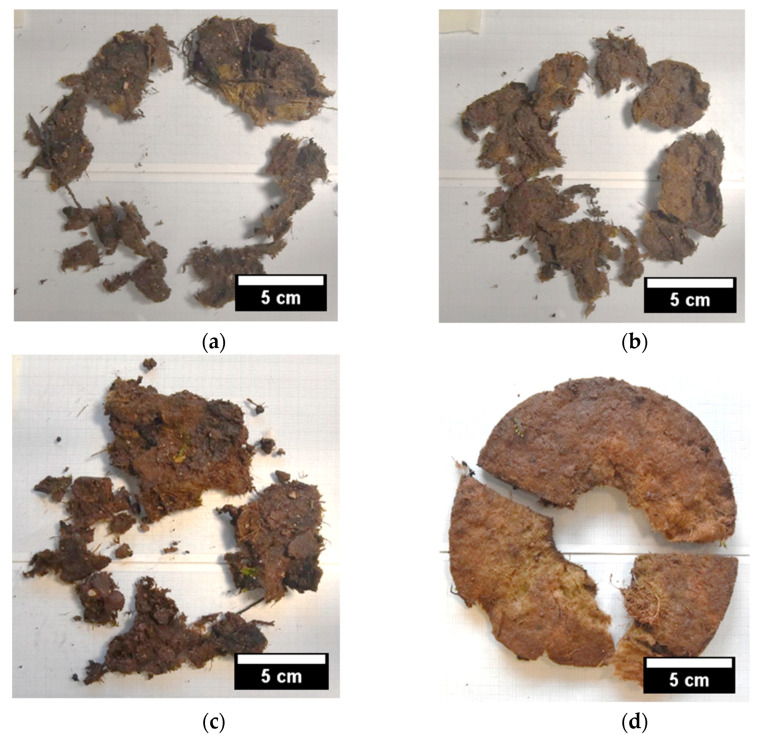
Representative images of mulching residues at the end of the planting test: (**a**) TSC_XG_CA60, (**b**) TSC_XG_CA60_cas, (**c**) TSC_XG_TA5 and (**d**) TSC_GEL.

Table 4 indicates that both samples cross-linked with TA (e.g., TSC_XG_TA5 and TSC_GEL) had higher residual mass with a significative difference (*p* < 0.05) compared to the TSCs based on XG cross-linked with CA. However, looking at the images of the residues shown in Figure 3a–d, there is a clear difference between the XG- and GEL-based samples.

Indeed, GEL-based TSCs (Figure 3d) still retained their structural cohesion, while all the XG samples disintegrated, indicating that they were subjected to more intense biodegradation in the soil. This difference could be explained by the greater density and thickness of GEL-based samples, which meant that they had more a stable and durable structure when applied in the field. In addition, the fact that XG has a higher water absorption capacity than GEL, and therefore a higher swelling degree, means that the XG-based TSC samples were subjected to repeated swelling/shrinking stresses during rainfall and subsequent drying periods. Frequent rainfall, particularly in the early stages of the experiment, may have compromised the effectiveness of the XG-TSCs, leading to the long-term disintegration of these samples. These different behaviours suggest the suitability of different applications for the two products: XG-based TSCs are suited for short-term applications (<1 year), such as in nurseries, and GEL-based TSCs are suited long-term applications (>1 year).

The observations performed on soil treated with SCs (not reported for the sake of brevity) did not allow for the identification of any residual particles, either because the material completely degraded in the soil or was incorporated within the soil structure. Further testing will be carried out in controlled environments to assess the biodegradability of the products and compare them with commercial non-biodegradable materials.

## 3. Materials and Methods

### 3.1. Materials

Commercial xanthan gum (XG) was provided by Galeno Srl (Prato, Italy) with a purity >91%, and a molecular weight (MW) of 1·10^6^ g/mol. GELITA IMAGEL^®^ LB, a type B gelatine powder with Bloom 113 (gel) and a viscosity of 2.29 mPa·s (6.67%, 60 °C), was purchased from GELITA AG (Eberbach, Germany). STEICO^®^ Flex-036 wood fibres, with an aspect ratio of 22.5–75 mm/mm and a bulk density of 60 kg/m^3^, were provided by STEICO SE (Feldkirchen, Germany). Natural cellulose fibres (Arbocel grade R) were kindly supplied by J. Rettenmaier & Söhne Gmbh (Rosenberg, Germany) in powder form with a cellulose content >99%, an average fibre length of 200–300 µm and a bulk density of 60–105 g/L. Vegetable glycerol (or glycerine), with a purity >98% and a MW of 92.1 g/mol, was produced by Farmalabor srl (Assago, Italy) and used as a plasticising agent. Citric acid monohydrate (CA), with a purity of 99.5% and MW of 210.14 g/mol, was supplied by Riedel-de Haën GmbH (Seelze, Germany) and used as a cross-linking agent. Tannic acid chestnut powder agent (TA), with a purity = 97% and MW of 1701.19 g/mol, was obtained from W. Ulrich GmbH (Eresing, Germany) and used as a cross-linking agent. Casein, with a MW of 23700 g/mol, was supplied by Thermo Fisher Scientific Inc. (Waltham, MA, USA) and used in solution as a protective coating. Sodium hydroxide (NaOH) was purchased from WHC GmbH (Schweitenkirchen, Germany) and used in the form of microbeads for pH adjustment. In addition, commercial SC made of potassium polyacrylate, i.e., Be-Grow Boost M, provided by University of Freiburg (Germany) and produced by Be-Grow GmbH (Neustadt an der Weinstraße, Germany), was used as a benchmark.

### 3.2. Sample Preparation

XG-based soil conditioner (SC_XG) was produced according to the methodology reported by Sorze et al. [35]. XG powder and Arbocel^®^ cellulose fibres were initially mixed in a 2:1 ratio. Then, hot water (60 °C) was added and mixed with an industrial mixer (Fama Industries Srl, Italy) until a homogeneous solution without lumps was obtained (5 min). Finally, the mixture produced was dried at room temperature and then milled using a Piovan^®^ RN166/1 granulator (Piovan SpA, Venice, Italy) to obtain a fine powder with a granulometry of 1–2 mm.

XG-based topsoil covers were developed according to the description reported in previous authors’ work [35]. Glycerine and XG were mixed in a 1.2:1 weight ratio, a then hot water (60 °C) was added and mixed until a homogeneous solution was achieved (3 min). WFs were subsequently added to the compound, which was further mixed until homogeneity was reached. Citric acid (60 wt% of XG) and tannic acid (5 wt% of XG) were finally added as crosslinking agents to produce two different compositions (TSC_XG_CA60 and TSC_XG_TA5). The samples were then manually spread in the form of discs with a diameter of 160 mm, dried at room temperature and thermally treated at 165 °C for 3.5 min in an oven to allow for the cross-linking reaction of XG and WFs with CA. A third sample was produced using the previous samples cross-linked with CA and applying a top-side protective layer consisting of an alkaline solution of casein before the field test (TSC_XG_CA60_cas). The casein coating was prepared according to the methodology described by Picchio et al. [65].

GEL-based mulching films (TSC_GEL) were prepared starting from two different batches (A and B): batch A was composed of 12 wt% of the total amount of water, while batch B was composed of 88 wt%. Initially, NaOH was added to batches A and B to ensure they had a pH of 9; then, TA was added to batch A at room temperature and GEL was added to batch B, pre-heated at 55 °C. The mixing process continued for 120 min until complete swelling of GEL in batch B was achieved; NaOH was added to keep the pH at 9. Batch A was then gradually added to batch B under continuous mixing (pH was kept at 9), followed by the addition of WFs. Finally, the homogeneous solution was poured into a mould and cured under an air flow for two weeks at room temperature.

Table 5 reports the different SC and TSC compositions used in the tomato planting trials.

### 3.3. Planting Experiment

The planting experiment took place in a garden located by the Department of Industrial Engineering of the University of Trento (46.06° N, 11.15° E, altitude 398 asl). A total of 72 tomatoes seedlings (*Solanum lycopersicum var. cerasiforme*) purchased from Consorzio Agrario Trento (Trento, Italy) were planted in 3 rows of 24 plants each (3 replications of each treatment per row). Plants with an initial height of 90 mm were transplanted from their pots by hand with a plant spacing of 400 mm. The experimental design was a randomised block design with eight treatments and nine replications (see Figure 3a). The treatments consisted in the application of the different SCs and TSCs described in Table 5. A control sample (i.e., not-treated plants) was also prepared for comparison. For the XG-based SCs (coded as SC_XG), two different treatments were applied: the first (coded as SC_XGa) was performed by mixing the product with the soil used to fill the plant pit after transplantation, while the second (coded as SC_XGb) by putting the product at the base of the plant pit before transplantation. In both cases, the amount of SC used was 15 g per plant. This quantity was chosen after preliminary studies and analyses. The commercial SC (SC_BeGrow) was used according to the supplier instructions, and 5 g of product was thus applied at the base of the plant pit before transplantation.

**Figure 4:**
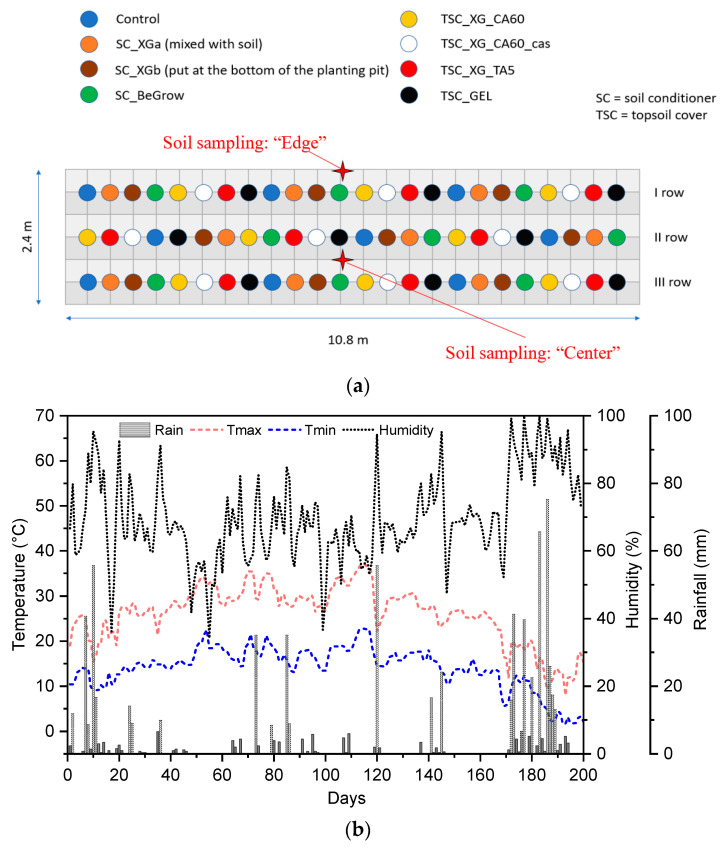
(**a**) Representation of the experimental design of the tomato planting trial. The blue dots represent the untreated tomato plants, while the other colours represent the tomato plants treated with TSCs or SCs. (**b**) Meteorological pattern recorded during open field experiment.

Relative humidity (RH%), rainfall, maximum and minimum air temperature were recorded daily throughout all the duration of the test using a Rotronic HL-1D (Rotronic AG, Bassersdorf, Switzerland) data logger located nearby the experimental field (see Figure 4b).

The experiment started in May 2023 and lasted until December 2023, when all the plants wilted. Before and after transplanting, no fertiliser was added to the plants and the experiment was performed without irrigation, as the water supply was only provided by rainfall.

As shown in Figure 4a, soil sampling was performed by Fondazione Edmund Mach (S. Michele all’Adige, Italy) at the border (close to the first row of plants) and in the centre of the planting area (between second and third rows of plants). The composition of the soil in the central part and at the edges of planting zone is reported in Table 6.

**Table 6:** Soil analysis results for samples taken from the edge and the centre of the planting zone.

Determination	Edge	Centre
Sand (2.0–0.05 mm)	412 g/kg	405 g/kg
Silt (0.05–0.002 mm)	458 g/kg	465 g/kg
Clay (<0.002 mm)	130 g/kg	130 g/kg
pH (in water ratio 1:2.5)	8.1	8.4
Total limestone	349 g/kg CaCO_3_	345 g/kg CaCO_3_
Active limestone	15 g/kg CaCO_3_	20 g/kg CaCO_3_
Organic substance	33 g/kg	12 g/kg
Assimilable phosphorus	27 mg/kg P_2_O_5_	15 mg/kg P_2_O_5_
Potassium	166 mg/kg K_2_O	114 mg/kg K_2_O
Magnesium	317 mg/kg MgO	284 mg/kg MgO

These tests revealed an overall loamy, alkaline, very calcareous soil with a good content of organic matter, potassium and magnesium, while the phosphorus concentration was limited. Moreover, both organic matter and soil nutrients (phosphorous, potassium and magnesium) were more concentrated at the edge of planting zone.

### 3.4. Evaluation of Yield Characteristics

The crop was hand-harvested when the ripe fruit rate reached about 90% (red stage). At harvest, the total fruit yield (ton/ha) and the number of total fruits grown per plant (NFT) were determined considering only red and disease-free fresh fruits.

### 3.5. Physico-Chemical Analysis of Tomato Fruits

#### 3.5.1. Evaluation of the Dry Matter

Dry matter content was determined by drying homogenised tomato samples in a conventional oven at 60 °C until a constant weight was reached. The dry matter content (%) was then calculated by dividing the final dry weight by the initial weight.

#### 3.5.2. Evaluation of the Brix Degree

Briefly, 20 g of tomatoes were blended homogeneously using a commercial blender. The final product was centrifuged with a 380R centrifuge (10 min, 4100 g-force; Hettich, Tuttlingen, Germany) in 50 mL conical tubes (SARSTEDT, Numbrecht, Germany). A few drops of supernatant were placed into the refractometer, RFM330-M (BELLINGHAM & STANLEY, Tunbridge Wells, Kent, UK), for a triple reading of the Brix degree.

#### 3.5.3. Evaluation of Lutein, Lycopene and β-Carotene Content

An amount of 0.5 g of the blended tomato samples was weighed into a 50 mL conical tube covered with aluminium foil, and 100 mg of butylated hydroxytoluene (BHT, Merck KGaA, Darmstadt, Germany) and 0.2 mg/L of astaxanthin (Internal Standard, >97%; Merck KGaA, Darmstadt, Germany) were added. The sample was dissolved by adding 2 mL of acetone; then, it was sonicated with an ultrasonic probe, UP 50h (Hielscher Ultrasonics GmbH, Teltow, Germany), for 1 min and then vortexed for 5 min with Vortex Multireax (Heidolph Instruments GmbH & Co. KG, Schwabach, Germany). Subsequently, 2 mL of hexane were added to the solution, and it was sonicated again for 30 s. The sample was vortexed for another 5 min, placed in an ultrasonic bath, LABSONIC LBS 1-6 (Flac Instruments, Treviglio, BG, Italia), for 5 min, and then centrifuged for 10 min (4100 g-force; Hettich, Tuttlingen, Germany). The supernatant was separated into a 15 mL conical tube; a second extraction was performed with hexane (2 mL) following the same procedure, and the products were collected in a second 15 mL Falcon tube. An amount of 1 mL from each extraction was filtered through a PTFE membrane and transferred into an HPLC vial for analysis.

Quantification of the different studied parameters was performed using an UHPLC-HQOMS (Ultra-High-Performance Liquid Chromatography–Hybrid Quadrupole Orbitrap Mass Spectrometer) system, consisting of Thermo UltiMate™ R 3000 HPLC (Thermo Scientific, Sunnyvale, CA, USA) coupled through an APCI source to a Q-Exactive™ high-resolution mass spectrometer (Thermo Scientific, Bremen, Germany). Chromatographic separation was achieved using a Develosil RPAQUEOUS C30 column, 2.0 × 150 (NW) measuring 3 μm (Nomura Chemical, Seto, Japan). Mass spectra were acquired in Full-MS/dd-MS2 mode, with respective resolutions of 70,000 FWHM (*m*/*z* 200, 1.5 Hz; scan range m/z 100–700) for Full-MS and 17,500 FWHM (12 Hz) for dd-MS2. Molecules were ionised in positive mode with the capillary temperature set at 250 °C, while sheath gas and auxiliary gas flows were set to 40 and 10 arbitrary units, respectively, with the probe temperature at 350 °C. The spray voltage was set to 5 kV. Matrix calibration was performed from 0.02 to 10 mg/L with pure standards supplied from Merck (Lutein, >92%; beta-carotene, >97%; Lycopene, >97%).

#### 3.5.4. Evaluation of Potassium Content

An aliquot of 500 mg of the homogenised fruit sample was weighed into a quartz tube (Milestone, Shelton, CT, USA), and 4 mL of ultrapure nitric acid was added. Samples underwent microwave digestion in a single reaction chamber (Ultrawave Milestone), following a three-step temperature programme [66]. After cooling, the solution was transferred into a polypropylene tube and diluted 3 times with ultrapure water prior to analysis. In each batch, a blank sample (i.e., ultrapure water) was prepared. Samples were analysed by Inductively Coupled Plasma–Optical Emission Spectrometry (ICP-OES; Perkin Elmer Optima 8300). Potassium (certified standard solution, 10 g/L) was purchased from CPI International (Santa Rosa, CA, USA), while yttrium (100 μg/L; Merck, Darmstadt, Germany) was used as an internal standard to correct matrix effects and instrumental drift [67].

### 3.6. Evaluation of Mulching Residues

At the end of the experiment, the plants were uprooted and the different analyses were conducted on the root system. Specifically, the root length and the stem diameter, measured at the same depth for each plant, were evaluated using digital callipers. Moreover, the root:shoot ratio, i.e., the ratio of belowground biomass to aboveground biomass, was also calculated. The water content of the roots was also measured, by taking the weight of the roots just taken out of the soil and after complete drying. In addition, a visual qualitative assessment of the characteristics of the root system, recorded through photographs, was performed.

### 3.7. Evaluation of Plant Root System

At the end of the experiments, the samples’ residues were collected, washed gently to remove the attached soil, dried, and weighed to calculate the residual mass of the samples. The residual mass (*M_residual_*) was calculated according to Equation (1):

(1) $$M_{residual}=\frac{M_{f}}{M_{0}}\cdot 100$$

where *M*_0_ and *M_f_* are the weights of the mulching films at the beginning and at the end of the test, respectively. In addition, a visual qualitative assessment of the characteristics of the mulching residues, recorded through photographs, was performed.

### 3.8. Statistical Analysis

All the experimental data are presented as the mean ± standard error of the mean. The data showed no normal distribution; therefore, the non-parametric Kruskal–Wallis method was used to highlight significant differences (α = 0.05) between the different treatments. Pairwise differences between treatments were assessed using Dunn’s post hoc test.

## 4. Conclusions

This work demonstrated the potential of bio-based and biodegradable xanthan- and gelatine-based composites as environmentally sustainable alternatives to conventional synthetic plastic mulching films and soil conditioners. Specifically, these materials were applied on a tomato planting trial under no irrigating conditions. Harvest results from the crop trial showed that, overall, the GEL-based TSC was a very effective treatment, increasing the total fruit yield by 50% more than the control. XG-based treatments improved crop yield when applied to soil with higher organic matter content. In addition, the presence of TSCs and SCs with water-retaining properties was found to extend the plant production period, thus increasing the total fruit yield of plants even under drought conditions, compared to the control. The physico-chemical analysis of the fruits showed that the treatments applied did not have a negative effect on the quality of the fruits. The qualitative evaluation of the plant root systems showed that, in the presence of high soil organic matter, most treated plants were characterised by a greater volume of fine roots than the control sample. Despite its limited scale, this study proved the feasibility of the concept of developing scalable alternatives to conventional synthetic plastic mulches and SCs, with the potential to reduce agricultural plastic waste and improve water use efficiency. One potential barrier to scaling up these materials is the high cost of biopolymers (xanthan and gelatine) and the energy-intensive production of these composites. However, the growing global output of biopolymers is expected to improve the economic viability of this system. For example, the material cost of xanthan gum for soil treatment has dropped from USD 250 to just USD 28 over the past three decades. Moreover, a 20% increase in the yield could be sufficient to offset the high production costs of materials. Future work should investigate long-term soil impacts, quantify life cycle benefits, and assess the economic scalability of these materials under different field conditions. Furthermore, more planting experiments will be conducted to investigate the effect of the produced materials on different crop types, climates and soil conditions. Specifically, tests will be carried out on crops that are sensitive to drought and water scarcity, such as cucurbitaceous species (watermelons, pumpkins and cucumbers) and leafy greens (spinach and lettuce).

## Figures and Tables

**Table 1:** Total yield, number of total fruits grown per plant and relative yield for each row.

Sample	Total Yield (Ton/ha)	NFT (Number/Plant)	Relative Yield I Row (%)	Relative Yield II Row (%)	Relative Yield III Row (%)
Control	19.1 ± 9.0	94 ± 32	-	-	-
SC_XGa	16.7 ± 13.9	87 ± 67	+17.8	−61.8	−20.1
SC_XGb	20.0 ± 15.1	98 ± 63	+33.9	−47.2	+6.5
SC_BeGrow	22.8 ± 18.2	108 ± 77	+56.3	−23.2	−22.6
TSC_XG_CA60	15.0 ± 12.7	79 ± 65	+6.3	−60.2	−42.1
TSC_XG_CA60_cas	15.7 ± 11.9	72 ± 47	+5.2	−60.5	−12.7
TSC_XG_TA5	10.6 ± 5.9	57 ± 22	−39.8	−49.3	−51.8
TSC_GEL	28.1 ± 14.1	130 ± 54	+55.4	−4.9	+121.8

**Table 2:** Physico-chemical characteristics of the fruits obtained from the different plants.

Sample	Dry Matter Content (%)	Brix Degree (°)	Lycopene Content (mg/kg)	β-Carotene Content (mg/kg)	Lutein Content (mg/kg)	Potassium Content (mg/kg)
Control	8.3 ± 0.5	7.32 ± 0.31	193.7 ± 72.3	97.3 ± 37.4	8.3 ± 2.1	3092.7 ± 45.4
SC_XGa	7.7 ± 1.8	6.93 ± 0.20	165.0 ± 22.5	138.7 ± 11.8	11.5 ± 1.8	3158.3 ± 93.7
SC_XGb	8.1 ± 0.8	6.93 ± 0.06	152.7 ± 16.9	137.0 ± 40.6	10.8 ± 2.5	3069.7 ± 296.9
SC_BeGrow	8.1 ± 1.0	6.98 ± 0.60	143.0 ± 18.9	134.0 ± 26.9	13.4 ± 1.1	2884.0 ± 204.7
TSC_XG_CA60	7.9 ± 1.4	6.53 ± 0.89	145.0 ± 21.3	121.0 ± 13.1	12.3 ± 1.1	2828.3 ± 433.3
TSC_XG_CA60_cas	8.1 ± 0.9	6.95 ± 0.28	179.3 ± 33.1	122.3 ± 42.8	12.3 ± 2.1	2964.0 ± 224.2
TSC_XG_TA5	8.3 ± 0.1	6.97 ± 0.05	207.7 ± 82.7	105.0 ± 9.9	10.6 ± 0.8	3028.7 ± 168.3
TSC_GEL	8.2 ± 2.6	7.12 ± 0.16	178.0 ± 63.6	111.7 ± 28.8	8.0 ± 0.7	3244.3 ± 173.5

**Table 3:** Results of the investigation of the plant root system of the tomato plants.

Sample	Root Length (cm)	Stem Diameter (cm)	Root: Shoot Ratio	Root Water Content (%)
Control	32.6 ± 4.2	1.0 ± 0.3	0.27 ± 0.03	80.9 ± 0.8
SC_XGa	34.8 ± 5.9	1.0 ± 0.2	0.31 ± 0.06	77.8 ± 2.4
SC_XGb	35.2 ± 3.7	1.0 ± 0.3	0.41 ± 0.11	79.7 ± 1.8
SC_BeGrow	34.1 ± 5.6	1.0 ± 0.1	0.36 ± 0.17	77.6 ± 2.0
TSC_XG_CA60	35.5 ± 6.7	0.9 ± 0.2	0.41 ± 0.16	80.5 ± 1.2
TSC_XG_CA60_cas	32.9 ± 3.9	0.8 ± 0.2	0.36 ± 0.10	78.8 ± 1.9
TSC_XG_TA5	34.7 ± 2.0	0.8 ± 0.1	0.35 ± 0.08	79.1 ± 2.1
TSC_GEL	33.9 ± 1.5	1.0 ± 0.1	0.36 ± 0.10	76.8 ± 1.8

**Table 4:** Residual mass values of TSCs at the end of the test.

Sample	M_residual_ (%)
TSC_XG_CA60	36.4 ± 6.7 ^a^
TSC_XG_CA60_cas	29.1 ± 12.1 ^a^
TSC_XG_TA5	57.6 ± 8.9 ^b^
TSC_GEL	56.3 ± 8.1 ^b^

Different letters indicate that the results are statistically different (*p* < 0.05) (see Section 3.8).

**Table 5:** List of produced samples used in the tomato planting trials.

Code	Composition	Characteristics
SC_XG	Soil conditioner based on xanthan gum and Arbocel^®^ cellulose fibre	Powder of 1–2 mm granulometry
SC_BeGrow	Commercial soil conditioner based on potassium polyacrylate	Powder of 100 µm granulometry
TSC_XG_CA60	Mulching film based on xanthan gum and STEICO wood fibres cross-linked with citric acid	Disc of 160 mm diameter and 3 mm thickness (areal density 900 g/m^2^)
TSC_XG_CA60_cas	Mulching film based on xanthan gum and STEICO wood fibres cross-linked with citric acid and coated with casein	Disc of 160 mm diameter and 3 mm thickness (areal density 900 g/m^2^)
TSC_XG_TA5	Mulching film based on xanthan gum and STEICO wood fibres cross-linked with tannic acid	Disc of 160 mm diameter and 3 mm thickness (areal density 900 g/m^2^)
TSC_GEL	Mulching film based on gelatine and STEICO wood fibres cross-linked with tannic acid	Disc of 160 mm diameter and 8 mm thickness (areal density 2400 g/m^2^)

## Data Availability

The raw data supporting the conclusions of this article will be made available by the authors on request.

## Abbreviations

The following abbreviations are used in this manuscript:

TSC	Topsoil cover
SC	Soil conditioner
XG	Xanthan gum
CA	Citric acid
TA	Tannic acid
GEL	Gelatine
WF	Wood Fibres
NFT	Number fruit grown per plant
